# 

**Published:** 1857-02

**Authors:** 


*Fifth Annual Meeting of the Mich. State Medical Society.*—In accordance with the resolution passed at the last meeting of the above society, that the next meeting be held on the day preceding commencement, the next annual meeting (5th) will be held in the college building, at Ann Arbor, March 24th, at 10 o'clock, A. M.

E. P. Christian, Secretary.

